# From Face-to-Face to Facebook: Probing the Effects of Passive Consumption on Interpersonal Attraction

**DOI:** 10.3389/fpsyg.2018.01163

**Published:** 2018-07-10

**Authors:** Amy C. Orben, Augustin Mutak, Fabian Dablander, Marlene Hecht, Jakub M. Krawiec, Natália Valkovičová, Daina Kosīte

**Affiliations:** ^1^Department of Experimental Psychology, University of Oxford, Oxford, United Kingdom; ^2^Department of Psychology, Faculty of Humanities and Social Sciences, University of Zagreb, Zagreb, Croatia; ^3^Department of Psychological Methods, University of Amsterdam, Amsterdam, Netherlands; ^4^Department of Psychology, Ludwig Maximilian University of Munich, Munich, Germany; ^5^Department of Psychology, University of Social Sciences and Humanities, Warsaw, Poland; ^6^Department of Psychology, Faculty of Social Studies, Masaryk University, Brno, Czechia; ^7^Behaviour and Health Research Unit, University of Cambridge, Cambridge, United Kingdom

**Keywords:** Facebook, passive consumption, homophily, social networking sites, passive use, longitudinal studies, interpersonal attraction

## Abstract

Social media is radically altering the human social landscape. Before the internet era, human interaction consisted chiefly of direct and reciprocal contact, yet with the rise of social media, the passive consumption of other users’ information is becoming an increasingly popular pastime. Passive consumption occurs when a user reads the posts of another user without interacting with them in any way. Previous studies suggest that people feel more connected to an artificial person after passively consuming their Facebook posts. This finding could help explain how relationships develop during passive consumption and what motivates this kind of social media use. This protocol proposes two studies that would make both a methodological and a theoretical contribution to the field of social media research. Both studies investigate the influence of passive consumption on changes in interpersonal attraction. The first study tests whether screenshots, which are widely used in present research, can be used as a proxy for real Facebook use. It measures the changes in interpersonal attraction after passive consumption of either a screenshot, an artificial *in situ* profile, or an acquaintance’s real Facebook profile. The second study relies on traditional theories of relationship formation and motivation to investigate which variables (perceived intimacy, perceived frequency of posts, perceived variety of post topics, attributional confidence, and homophily) moderate the link between interpersonal attraction before and after passive consumption. The results of the first study provide insights into the generalizability of the effect by using different stimuli, while also providing a valuable investigation into a commonly used method in the research field. The results of the second study supplement researchers’ understanding of the pathways linking passive use and interpersonal attraction, giving the field further insight into whether theories about offline relationship formation can be used in an online context. Taken together, this protocol aims to shed light on the intricate relation between passive consumption and interpersonal attraction, and variables moderating this effect.

## Introduction

1.32 billion people use Facebook daily, each spending about 50 min on the site (Nowak and Guillermo, 2017). That sums up to 125 years of Facebook use – every day. This trend to use social media, which has spread around the world, shows no signs of slowing down. In the meantime, it has not only changed how we spend our time, it has also introduced many new communicative features to our social life.

While social media use is often treated as a monolithic concept, it supports several different behaviors. Burke et al. (2010) identified two distinct types of social media use: directed communication and passive consumption. Directed communication does not only consist of chatting one-on-one, but also includes tagging in photos, commenting, or writing on profiles. In contrast, passive consumption includes browsing News feeds and scrolling through others’ posts and public conversations without direct interaction (Schlosser, 2005).

It is easy to equate social media use to the former activity, interpreting it as a way to socialize directly with others. An interesting trend has, however, emerged over the last years. In 2012, 70% of Facebook users were active users, spending their time posting statuses or chatting with friends. This number decreased to only 52% in 2014 (McGrath, 2015), while the time spent passively consuming markedly increased. Recent research suggests that passive Facebook use (Brandtzæg, 2012) – when users spend most of their time viewing others’ content while not creating content of their own (Burke et al., 2011) – is now the norm (Gerson et al., 2017).

### Passive Use and Why It Is Interesting

It is, however, still unclear how passive consumption fits into our previous interpersonal social landscape. Trying to understand this, many research projects have focused on whether passive consumption can increase social connection and interpersonal attraction (e.g., Utz, 2015; Orben and Dunbar, 2017). Yet the evidence for whether passive consumption on social media can support relationship development is still sparse and mixed (Frost and Rickwood, 2017). While it is not as intimate as one-on-one conversations, some suggest passive consumption helps maintain broader social networks (Burke et al., 2011). Passive consumption of friends’ and acquaintances’ posts on the News feed gives users access to a much larger social circle, letting them overcome social and time barriers that make direct contact laborious or impossible (Lewis and West, 2009; Burke et al., 2011). Passive consumption can also positively influence emotional well-being and provide informational and emotional support (Ballantine and Stephenson, 2011; Good et al., 2013).

Yet studies also demonstrate that passive use of social media has potential negative effects. These negative consequences include increased loneliness, undermined affective well-being, raised social anxiety, and envy (Burke et al., 2010; Krasnova et al., 2013; Verduyn et al., 2017). Passive use of social networking sites also promotes weak and low-commitment ties (Lewis and West, 2009), social bonds that are not as intimate as the ones formed via direct interactions. These unique effects make it apparent that passive consumption is not identical to offline interpersonal interaction.

Nevertheless, increasing proportions of the population are using Facebook passively and it is therefore important to research what mechanisms determine its effect on interpersonal attraction (Steers, 2015). Previous psychological theories – put forth to explain the development of interpersonal attraction in the offline world – can be a helpful guide in that process. These theories suggest a wealth of possible moderators of face-to-face communication which affect interpersonal attraction. By investigating whether these moderators also influence changes in interpersonal attraction during passive consumption, researchers could take substantial steps toward a more comprehensive picture of how passive consumption affects social attraction. Our protocol details two studies that take first steps in this direction.

### The Proposed Studies

The two proposed studies aim to make both a methodological and a theoretical contribution to the field of social media research. The first study examines the novel experimental manipulation we intend to use in our subsequent study. In this first study, we test whether previous manipulations used in research are valid proxies of social media use, and whether our manipulation offers an improvement. Instead of having participants passively consume directly on Facebook, previous research has often used screenshots of posts as a proxy. However, the extent to which this substitution is sound has not been assessed before, thus previous results might have been artifacts of the experimental manipulation. We examine this by using different mock-ups of Facebook profiles in the first study, and comparing them to actual passive consumption on the platform. In particular, we want to measure the change in feelings of interpersonal attraction after passive consumption of either a screenshot, an artificial *in situ* profile, or an acquaintance’s real Facebook profile.

After exploring the effect of different Facebook mock-ups, we aim to investigate potential variables that might moderate the effects of passive consumption on feelings of interpersonal attraction in our second study. This study will allow us to integrate our work into the existing theoretical literature. Since social media research is a relatively new field, we use traditional psychological theories of relationship formation and motivation to form hypotheses about variables moderating the link between interpersonal attraction before and after passive consumption. We then use these moderators in our models, examining how they operate in a social media context. Doing so will provide clues about what theories might help researchers understand aspects of online sociality and relationship formation over social media in the future. Using a longitudinal design, we furthermore try to detect long-term changes of interpersonal attraction to add up to existing literature focusing mainly on short-term and cross-sectional effects of passive consumption.

#### Study 1: Comparing the Effect of Different Facebook Profile Mock-Ups

Many previous studies in the field of social media research have used screenshots of real or artificial posts (e.g., Bazarova, 2012; Lin and Utz, 2015; Rains and Brunner, 2015; Orben and Dunbar, 2017) to investigate cross-sectional changes in interpersonal attraction after passive consumption, despite this method not being formally validated. Our protocol aims to examine this widely used methodology using *in situ* Facebook profiles: fake profiles on the actual Facebook platform. We compare the effects of these *in situ* profiles on interpersonal attraction to the effects of screenshots and real Facebook profiles.

We further want to examine how the passive consumption of artificial profiles (screenshots or *in situ* profiles) affects the consumer’s interpersonal attraction toward the profile owner in comparison to passive consumption of real profiles. This will, on the one hand, allow us to test whether our *in situ* profile manipulation is valid, while also providing a valuable investigation into a commonly used method in the field.

Perceived physical attractiveness of the profile owner could influence the effects we are studying. For example, physically attractive people are liked more by others and, due to the halo effect, other characteristics like their personality or behavior are also rated more positively (Berscheid and Walster, 1974). The elevation in social attractiveness is higher for users that are perceived as more attractive.

However, we also predict that the effects of different profile mock-ups are dependent on the type of the outcome variable measured. For example, feelings of attraction take longer to develop and it is therefore possible that interpersonal attraction will not change after a single browsing session used in our study. On the other hand, certain processes, such as the formation of interpersonal evaluations, may unfold more quickly and therefore be impacted by the validity of manipulation by different profile mock-ups.

RQ1: Can screenshots or artificial *in situ* profiles be used as a proxy for real Facebook use?

As a similar study has not been previously completed, we do not have any strong predictions. However, as past research has heavily relied on screenshots, we tentatively suggest that there will be no differences in the increase in our dependent variable across all three conditions (screenshots, *in situ* profile and real profile). Thus, our hypotheses are that both screenshots and *in situ* profiles are not inferior to real profiles.

H1: (a) There will be no difference between post- and pre-test scores in interpersonal attractiveness and interpersonal evaluation for participants who view Facebook posts of an actual Facebook friend’s profile compared to participants who view screenshots of posts (Hypothesis 1a).
  (b) There will be no difference between post- and pre-test scores in interpersonal attractiveness and interpersonal evaluation for participants who view Facebook posts of an actual Facebook friend’s profile compared to participants who view posts of an artificial profile (Hypothesis 1b).

We further expect an increase in interpersonal attractiveness and interpersonal evaluation for all conditions after passively consuming Facebook posts.

#### Study 2: Investigating the Change in Interpersonal Attraction After Passive Consumption and the Potential Moderators of This Process

Many previous studies in the field of social media research have focused on short-term cross-sectional changes in interpersonal attraction (e.g., Bazarova, 2012; Lin and Utz, 2015; Rains and Brunner, 2015). We aim to extend this research by investigating potential moderators and by using a longitudinal study design. By setting up three real Facebook profiles that our study participants will add, and by posting on two of them on a regular basis, we aim to mimic real-life Facebook passive consumption in an experimental setting. One profile will not post during the study period, and thus serve as a control condition. This control condition will help us track changes back to the passive consumption of posts: the experimental manipulation.

Based on the previous research of passive consumption we hypothesize the following:

H2: Feelings of interpersonal attraction toward the Facebook profile owner will increase in the two experimental conditions (updated Facebook profiles), but not in the control condition (Facebook profiles without any new updates).

Generally, disclosure of personal information can help or hinder relationship development (Orben and Dunbar, 2017). This diversity in effects makes it important to examine which variables moderate how disclosure of personal information, either offline or online, affects interpersonal attraction. Several psychological theories have addressed this in offline interaction, examining how relationships are formed through face-to-face self-disclosure. While the theories were conceptualized to explain offline behavior, we believe that they could harbor important insights into how interpersonal attraction evolves after passive consumption.

In particular, theorists have highlighted many different factors surrounding self-disclosures that might facilitate the formation of a social bond. We can re-appropriate these factors and look at them in a social media context to examine the effects of passive consumption. Thereby, we find three theoretical approaches to be most helpful: Social Penetration Theory, Uncertainty Reduction Theory, and Homophily.

Social Penetration Theory highlights the importance of activities which involve intimate informational disclosures about oneself as the driving force of relationship development (Altman and Taylor, 1973). It suggests that relationships become more intimate over time as the members of a dyad intentionally disclose information about themselves. This process is gradual and starts with revealing only the superficial information about oneself. By continuously sharing more and more intimate information, however, individuals involved in dyadic communication develop closer relationships. As people like those who reveal more about themselves, liking increases accordingly (Altman and Taylor, 1973). Hereby, the breadth and depth of the disclosed information are crucial. The dimension is reflected by the intimacy of disclosures, whereas the breadth refers to the quantity of disclosures, reflected by the variety of topics and the frequency of messages disclosed. According to Social Penetration Theory, disclosure breadth and depth covary, because disclosing on a higher frequency and on a wide variety of topics typically reveals personal information.

Social Penetration Theory emphasizes the need for self-disclosures to be perceived as intimate and to cover a variety of topics for a social relationship to form. Based on this theory, one would predict that higher perceived intimacy and a greater perceived variety of Facebook posts, as well as a greater perceived frequency of posts, would lead to a higher elevation of interpersonal attraction over time.

RQ2: Does the perceived intimacy of posts moderate the change in interpersonal attractiveness after passive consumption?

RQ3: Does the perceived frequency of posts moderate the change in interpersonal attractiveness after passive consumption?

RQ4: Does the perceived variety of posts moderate the change in interpersonal attractiveness after passive consumption?

Uncertainty Reduction Theory (Berger and Calabrese, 1975) contributes another approach to explaining interpersonal attraction. It suggests that a reduction of uncertainty about another person supports the development of interpersonal attraction. To reduce uncertainty, individuals engage in activities that provide information. They can use strategies that are passive (i.e., observing others’ behavior), active (i.e., proactive information-seeking effort), or interactive (i.e., direct communication) (Berger and Calabrese, 1975). If a piece of information increases attributional confidence, that is, if people feel like they know each other and can predict each other’s behaviors, it should, therefore, increase feelings of connection (Antheunis et al., 2010). The theory, therefore, predicts that the more uncertainty is reduced by passive consumption, the stronger is the change in interpersonal attraction over time. Hereby, passive consumption serves as a form of passive behavior observation reducing uncertainty about the user.

RQ5: Does attributional confidence moderate the change in interpersonal attractiveness after passive consumption?

Additionally, homophily, or the perceived similarity with another person, may elicit liking and develop relationship formation (Currarini and Mengel, 2016). Shared interests, values, sociodemographic dimensions and other features of similarity impact how attracted a person feels to someone (Montoya et al., 2008). This is related to shared social identity, which makes people who are a part of one’s social group more favorable (McPherson et al., 2001). This in-group bias refers to “the systematic tendency to evaluate one’s own membership group (the in-group) or its members more favorably than a non-membership group (the out-group) or its members” (Hewstone et al., 2002, p. 576). Thus, if users feel like they share the profile owner’s interests or views after browsing through their Facebook posts, they might evaluate these users more favorably compared to the ones who they do not have any information about, and thus express more elevated levels of interpersonal attraction toward the profile owners. In contrast to Social Penetration Theory, which highlights the importance of intimate disclosure of a wide range of topics, and Uncertainty Reduction Theory emphasizing the need to reduce insecurity about the person disclosing information, the perceived similarity with the person disclosing information may be another crucial element for a relationship to form.

RQ6: Does homophily moderate the change in interpersonal attractiveness after passive consumption?

By examining the outlined moderators, which we derive from traditional psychological theory, we hope to contribute to a greater understanding of the underlying processes of relationship formation and development on social media. We are aware that there might be other variables of interest changing the level of interpersonal attractiveness, augmenting our moderators, and control variables. However, by selecting the most relevant theories in the field, we hope to cover the most important factors influencing interpersonal attractiveness, and to test whether traditional theories of relationship formation are applicable to online relationship formation.

## Methods

### Study 1: Examining the Effect in Real Life

#### Participants

Study 1 will take place in a lab. Participants will be recruited via a departmental mailing list, and participants need to be between 18 and 50 years old, proficient English speakers, and Facebook users. If the lab is in a different country from the United Kingdom or United States, we will translate the introduction and conclusion of the study, and we will recruit proficient English speakers so the rest of the study should proceed as normal in English. The participants will be paid a cash reimbursement for their participation.

#### Procedure

After entering the lab, participants are assigned to one of three conditions: the real Facebook profile, fake profile, or profile screenshots condition. In the study introduction, participants will be led to believe that the study examines their general attitudes toward Facebook. As we use deceptive elements in our study, we will ensure that the participants are debriefed extensively at the end of the study.

At the beginning of the study, participants will be presented with an information sheet and consent form. Participants will be told that they can withdraw from the study at any time. After providing basic demographic information, participants fill out a distractor questionnaire concerning their opinions about privacy, sharing news stories, and promoting hate speech on Facebook, in order to obfuscate the real purpose of the study and avoid biasing effects. Afterwards, participants will be asked to log into their Facebook account. Subsequently, participants will be presented with the top half of a Facebook profile in the artificial and real condition, or with a screenshot of the top half of a profile in the screenshot condition. The target’s Facebook profile will show a woman in her early 1920s along with basic demographic information, such as her alma mater, hometown, and employment. The profile will also contain a profile picture and a cover photo. Participants then rate how attracted they are to the person and evaluate the interpersonal characteristics of the person (for more information on measurements, see the section “Measures”).

Participants then browse through the first 10 posts of the respective Facebook profile (or look at 10 screenshots of Facebook posts). After scrolling through the posts/screenshots, the participants will again complete the measure of interpersonal social attraction, interpersonal evaluation, and the distractor measure. Subsequently, they will complete further measures about homophily, attributional confidence, perceived intimacy, and perceived valence of the posts, as well as physical attractiveness of the profile owner (McCroskey et al., 1975; Clatterbuck, 1979; Watson et al., 1988).

##### Artificial Facebook profile or screenshot condition

In the artificial Facebook profile condition or the screenshot condition, we use a selection of posts that were pretested to make sure they are perceived as highly appropriate and credible in order to avoid confounding effects. We additionally measured the posts’ perceived valence and intimacy to use these as control variables, i.e., to be able to track back potential changes to these variables if the effect is not merely caused by the change in interpersonal attraction. Generally, we ensure that our posts do not just include written text, but a variety of post types (e.g., photos, photos with text, written text, shared article, and video) in order to make the profile appear as real as possible. In the artificial Facebook profile condition, we combine these posts into a real Facebook profile on the website, whereas in the screenshot condition, we take screenshots of the posts and present them to participants one-by-one.

##### Real Facebook profile

In the real profile condition, participants will be asked to look through their friend list and choose a person whose profile they have not looked at during the last 2 months, who they feel like they do not know very well, and who they do not have any strong feelings about. Participants that do not have such a person in their friend’s list will be excluded from the study. Participants choosing a close friend might score higher on ratings of interpersonal attraction after passive consumption and thus confound our results. Our instructions and exclusion criteria should therefore diminish potential confounding effects that could arise from previous feelings of connectedness toward the profile owner.

The research assistant will ask the participants who the person they selected is and take notes about the relationship between the participants and the user. The participant is then asked to scroll down the profile looking at the first 10 posts only. As the fake Facebook profile includes 10 publicly visible posts, this will enable us to compare the subsequent measures between the two conditions.

#### Measures

##### Interpersonal Attractiveness Questionnaire (IAQ)

We will use the Interpersonal Attractiveness Questionnaire (IAQ) (Montoya and Horton, 2004) to measure participants’ perceived attractiveness of the profile owner. The questionnaire consists of nine items with the response format being a seven-point Likert-type scale, ranging from “I strongly disagree” to “I strongly agree.” By re-analyzing previous data, we found that the IAQ possesses favorable psychometric properties. A single-factor confirmatory factor analysis model has been fit to the data using the lavaan package (Rosseel, 2012) for R (R Core Team, 2017). Most fit indicators (CFI = 0.98, TLI = 0.97, PCLOSE = 0.08, normed χ^2^ = 3.31, SRMR = 0.02) suggest that the model achieves close fit. To assess reliability, tau-congeneric, tau-equivalent, and parallel versions of the model were calculated (Cho, 2016). Comparing the tau-congeneric and the tau-equivalent models using the χ^2^ difference test showed that the tau-congeneric model achieves better fit (χ_diff_^2^ = 84.9, *df*_diff_ = 8, *p* < 0.001). Reliability was calculated using the procedure for tau-congeneric models proposed by Raykov (1997). This analysis yielded a high reliability of 0.92.

##### Interpersonal evaluation inventory

To examine how participants evaluate social characteristics of the profile owner, which could evolve more rapidly than the feelings of attraction, we will use the interpersonal evaluation inventory (Kelly et al., 1980). The questionnaire consists of 24 adjectives (e.g., considerate, educated, and honest) and two question-like phrases describing or related to a person (in this case, the profile owner). The participant rates the person on a seven-point Likert-type scale adjusted to each adjective (e.g., “Extremely inconsiderate” to “Extremely considerate”). The scoring direction is determined randomly for each adjective. Previous research indicates that the Inventory is valid with four underlying factors. However, for the purpose of our study we have decided to only use the items from the Likeability subscale. Likeability subscale is the most relevant subscale for this study content wise and with its 14 items it composes the majority of the inventory. Using unifactorial measures will greatly simplify our analyses, with negligible information loss.

##### Proactive attributional confidence scale (PACS)

We will use the proactive attributional confidence scale (PACS) (Clatterbuck, 1979) to measure attributional confidence. The scale consists of seven items, with the response format being a six-point Likert-type scale ranging from “Not confident at all” to “Very confident.”

##### Attitude homophily scale (AHS)

The attitude homophily scale (AHS) (McCroskey et al., 1975) will be used to measure homophily. The scale consists of four items. Each item consists of two opposite claims (e.g., “Doesn’t think like me” and “Thinks like me”). These two claims are anchor points of a seven-point Likert-type scale which is used to indicate a participant’s response.

##### Distractor measures

To conceal the true purpose of the study, which could become evident if the participants are only completing social attraction/evaluation measures at two close timepoints, we will use a distractor measure to lead the participants to believe that the aim of the study is to measure their attitudes on Facebook. To achieve this, we designed a questionnaire with good face validity containing items such as “Facebook makes it easier to keep in touch with other people,” “The way Facebook is set up is a threat to privacy,” “Some Facebook users incite violence toward minorities using the network.” We believe that attitudes toward Facebook are a good distractor measure because the central activity of the experimental procedure is Facebook use. The content of the items was chosen with caution not to prime the participant to form a certain attitude toward the profile owner (e.g., we did not use any items related to Facebook addiction).

##### Control variables

As additional measures, we also include perceived intimacy, perceived valence, and perceived physical attractiveness. To assess the perceived intimacy of posts, participants will be asked to rate, how intimate the posts made by each of the profile owners were on a seven-point Likert scale from 1 (not at all intimate) to 7 (very intimate). Participants will be asked to rate the general valence on a seven-point Likert scale from 1 (very negative) to 7 (very positive). Perceived physical attractiveness of the profile owner will be assessed by a single item question “How physically attractive do you find this person?,” by rating from 1 (not attractive at all) to 7 (very attractive). Data on perceived intimacy and perceived valence will be collected only after the participant has scrolled through or saw screenshots of 10 Facebook posts.

#### Analysis

Statistical analysis will be carried out using the R programming language (R Core Team, 2017) and, together with the raw data, will be made publicly available on the Open Science Framework.

Our goal in Study 1 is to test whether artificial Facebook profiles and screenshots can be used as a proxy for real Facebook profiles. If so, we expect that there is no difference in the mean change on the dependent variable across these conditions. To test this, we employ a multiple-group latent difference model (McArdle, 2009, pp. 584–585; Allison, 1990) using the change in the mean interpersonal attractiveness scores and in the mean interpersonal evaluation scores as dependent variable, respectively. Since we cannot experimentally control for the content and type of the post in the real Facebook condition, we use perceived intimacy of posts, perceived valence of posts, and perceived physical attractiveness of the profile owner as control variables.

### Study 2: Investigating the Change in Interpersonal Attraction

#### Participants

We aim to recruit about 400 participants using a convenience sample over social media and through mailing lists. For inclusion in the study, participants need to be between 18 and 50 years old, proficient English speakers in order to understand study instructions and Facebook profile content, and Facebook users. Participants will get a chance to win a raffle prize upon completion of Study 2 for reimbursement. We will endeavor to collect all participants of Study 2 within 2 weeks.

#### Procedures

First, participants will receive an information sheet and a consent form via email. If they consent to participate in our study, participants will be asked to indicate their demographic data (age, gender, race, and location) and to log into their Facebook accounts. By following a link, they will be instructed to take a look at a profile of “another research participant” for 1 min. Participants will be able to see only the basic information about the profile owner – name, profile photo, and cover photo. Subsequently, participants will be asked to return to the survey platform to fill in questionnaires which measure interpersonal attraction toward the profile owner and perceived physical attractiveness of the profile owner.

The same procedure as outlined above will be repeated with two other Facebook profiles of “other research participants.” Participants will be instructed to be friend the Facebook profiles and will be informed that within 2 weeks, the three other research participants whose posts they saw will be instructed to accept the friend request. They will be asked to mark the new Facebook friends’ profiles as “see first,” which are an option on Facebook that ensures participants see all of the profiles’ posts in their News feeds, by putting these posts on top of all others. This will ensure that all participants are exposed to the posts, and that we can attribute changes in interpersonal attraction to the passive consumption. Participants will be able to see the new Facebook friends’ profiles and posts; however, they are not allowed to directly interact with them by messaging, commenting, or liking their posts.

The Facebook profiles will be set up prior to the study with the aim to make them seem realistic. To ensure this, we will remove the date our profile was created from the information box on the set-up Facebook profile. All three profiles will have 10 posts in their history. However, only two of the profiles will add status updates throughout the study period, i.e., be the experimental condition profiles. One profile will not add any new posts during the study period, i.e., be the control condition profile.

Participants will be sent a questionnaire after 2 weeks (Wave 2), 4 weeks (Wave 3), and a final questionnaire after 6 weeks (Wave 4) via email. In Wave 2, Wave 3, and Wave 4, they will be asked to fill in the interpersonal attraction measure and measures of control variables that will include, aside from (1) perceived physical attractiveness of the profile owner, (2) perceived intimacy of posts, (3) perceived frequency of posts, (4) perceived variety of post topics, (5) attributional confidence, (6) homophily, and (7) perceived valence of the posts. Next to the questions about perceived intimacy and valence of the posts, there will be the option “this person did not post during the last 2 weeks” that participants can select. At the end of Wave 4, after completion of the questionnaires, participants will be asked whether they remember three profile posts, only two of which were actually posted. This will serve as a manipulation check to test whether participants were exposed to the profile’s posts. Finally, participants will be asked to rate how credible the profile was overall, and will be debriefed about the real aim of the study.

#### Measures

Study 2 will include the same measures used in Study 1 (described in Section Measures), but not all of the measures will be used. Specifically, we will use the IAQ, PACS, AHS and measures of perceived intimacy, perceived valence, perceived physical attraction, and perceived frequency of posts. Participants will rate the perceived frequency of posts on a seven-point Likert scale from 1 (posts very rarely) to 7 (posts very often).

#### Analysis

Our goal in Study 2 is to explore whether theories of offline communication can inform online communication, particularly passive consumption. Concretely, we aim to test whether (1) perceived intimacy of posts, (2) perceived frequency of posts, (3) perceived variety of post topics, (4) attributional confidence, and (5) homophily influence the change in interpersonal attractiveness over time (see RQ2-6).

Our analysis uses structural equation modeling and proceeds as follows. As a prerequisite, we will first test whether the factor structure of interpersonal attractiveness is invariant across time; that is, we will test for strong measurement invariance (Little et al., 2007, pp. 358–359). First, to see whether our intervention, that is, participants passively consuming posts, was successful, we will compare the change in interpersonal attractiveness across the three conditions (see above) using a multiple-group latent growth-curve model (e.g., Newsom, 2015, chapter 7). We will also test whether the change can be adequately captured by a linear function. Second, to test whether the variables derived from theory influence the change in interpersonal attractiveness, we will include them as time-invariant covariates, controlling for perceived physical attractiveness of the profile owner, and perceived valence of the posts.

## Limitations And Impact

As we will implement a complex procedure in a relatively new field, our study has multiple limitations. However, we strongly believe that the insights acquired by running this study will outweigh these limitations. Our work will allow researchers to reassess traditional psychological theory and will provide a stepping stone in the use of more complex longitudinal and realistic methodology in social media research.

### General Limitations

Our sampling strategy might be criticized as participants will be recruited through social media or mailing lists and therefore might not be representative of the population. While true, we do not see an issue with this. As our study requires participants to be (frequent) social media users, our sample cannot be fully representative by intent and we can generalize our results only to social media users.

Participants may also guess the purpose of our study or they might know that the artificial profiles are fake. In Study 1, this could mean that using fake profiles is not a solid substitute for simulating real life Facebook use. However, in Study 2, this would be a cause of concern as we cannot generalize attraction developed toward a non-existent person to attraction developed toward a real person. Therefore, at the end of both studies, we will include a question about what the purpose of the study was, to find out whether any of the participants guessed the real aims of the experiment. In case they did, we will exclude these participants from the study.

### Limitations Study 1

One of the main limitations of the design of Study 1 is the lab setting. The lab environment is artificial and may lead to behavior that would not occur in a natural setting. The laboratory will, however, allow us to control that the participants focus solely on the study and do not digress to other websites. The artificial setting of Study 1 is not just limited to the laboratory setting, but also includes the task of scrolling through Facebook profiles themselves. Being asked to look through someone else’s profile for a set period of time might provoke a different kind of reaction than the one that is experienced while browsing through profiles voluntarily. Participants might, for example, speculate whether they chose the right person from their list of friends, which could decrease their focus. Therefore, we have to be careful when generalizing our results.

Another limitation is linked to the selection of the real Facebook profile. As the chosen profile owner is already acquainted with the participant, confounding variables due to previous feelings, or certain attitudes toward this particular person might impact our study in an unpredictable manner. We, however, try to control for these variables by telling the participants to choose a person whose profile they have not looked at in the last 2 months, who they feel like they do not know very well, and who they do not have any strong feelings about. We need to use a real person in order to be able to compare real Facebook consumption to simulated Facebook consumption used in social media research, and we therefore need to take these limitations into account.

In the screenshot conditions, participants do not scroll through the posts but instead view them one by one. While unlikely, it could be that scrolling affects the interpersonal attractiveness judgments of the participants. However, as previous studies also did not include scrolling in the screenshot conditions, we decided against it to be as close to previous studies as possible.

### Limitations Study 2

While developing the design of longitudinal studies, choosing the optimal lag between time points is critical (Gollob and Reichardt, 1987; Little, 2013). In this respect, there is a potential limitation of our study. First, our proposed time period might be an overestimate, meaning that interpersonal attractiveness might change more rapidly. However, it could also be an underestimate as the change in interpersonal attractiveness might be a long process which cannot be tracked during the span of our study. Overall, choosing the lag is difficult, especially in areas without precise theoretical guidance. We thus acknowledge this limitation fully.

With longitudinal studies, the loss to follow-up is a known threat. While we recognize this risk, we hope to reduce it by running a fairly short longitudinal study (6 weeks). We will also send reminders to fill out the questionnaires in each wave.

Another general limitation is that all study participants befriend three unbeknownst to them, young, Caucasian, female students, and receive their status updates. We can therefore, strictly speaking, generalize our conclusions only to these particular profiles; our stimulus sample size is *n* = 3 (Wells and Windschitl, 1999). This befriending of unknown (fake) profiles is also different from the typical Facebook dynamic, where users typically know persons they add to their friend list, have met them before or have mutual friends. While this might at first appear as a limitation, we will design the profile to be representative of a prototypical Facebook user, therefore our ability to generalize will not be limited severely. Previous studies in this area have used artificial profiles successfully and we believe that our improvement in methodology (conducting the study on Facebook) adds greatly to the existing literature. Furthermore, because we test whether our employed measures are invariant across the fake profile and the real profiles, we can, to some extent, empirically assess this generalizability problem.

### Impact

By completing the studies set forth in this proposal, we expect to shed further light on the motivational background and outcomes of passive consumption. The trend toward spending time online passively consuming has developed for several years, yet it has not been extensively researched. To gain further understanding about this process, we aim to utilize traditional social psychological theory. Can classic theories of relationship formation be applied to our online use of social media? If so, they can help us explain the effects in more detail and give us further insight into whether interpersonal relationships can be formed solely by passively consuming someone’s online information. By examining variables derived from traditional psychological theory, we can also test what might be moderating effects of passive consumption and whether these moderators are similar to those influencing face-to-face communication. This will allow us to highlight theories that could be utilized by social media researchers in the future.

Our protocol also contributes to the field methodologically. Using a longitudinal design with *in situ* artificial profiles that will be active throughout the duration of study instead of screenshots of posts and profiles, we take a novel and innovative step toward highlighting drivers and outcomes of passive consumption. Furthermore, our study is one of the first to examine whether the artificial passive consumption paradigm (participants looking at a stranger’s profile) is a good simulation of actual passive consumption. It combines both real and fake Facebook profiles as well as screenshots of posts that have been used in social media research up until now, to inspect whether we can apply previous studies which used an artificial environment (e.g., Deters and Mehl, 2013; Orben and Dunbar, 2017) to explain real-life Facebook use. It is not known whether these widely used results are solely an artifact of the research paradigm because comparable validity checks have not been conducted yet. By conducting our research, we can, therefore, possibly increase the validity of methodological approaches in future studies of social networks. Thus, our proposed studies are an important addition to social media research in multiple different ways.

## Conclusion

With social media constantly growing in popularity and becoming indispensable in the lives of the majority of people in developed countries, it is important to study the drivers and impacts of its use. In this study, we focus on passive consumption on social media as it has overtaken active use in overall popularity over the past years (Gerson et al., 2017). The reasons behind this trend, and its impact on social life, have not been thoroughly researched. Previously, passive use has been described as lacking the reciprocity and depth to aid form social connections. However, there has been evidence that passive use might help us feel more connected which we aim to investigate further. Using two studies, we want to take a critical look at the methodology used in the social media research area, by comparing previously used screenshots of fake profiles to actual fake profiles on Facebook and to real profiles, while also examining possible changes in interpersonal attraction after passive consumption on Facebook, as well as possible factors influencing this change. By conducting this research, we hope to answer important questions regarding social media use and bring the research field closer to understanding these phenomena.

## Ethics Statement

The study materials and consent forms have been developed in accordance with ethical norms and guidelines from all participating institutions. The study received approval by the Central University Research Ethics Committee (CUREC) at the University of Oxford.

## Author Contributions

AO conceived the idea for this project. AO, AM, FD, MH, JK, NV, and DK have made substantial intellectual contribution to this work. All authors gave the publishing approval.

## Conflict of Interest Statement

The authors declare that the research was conducted in the absence of any commercial or financial relationships that could be construed as a potential conflict of interest. The reviewer MEJK declared a shared affiliation, with no collaboration, with one of the authors, AO, to the handling Editor.
